# Supplementary material for: Predicting customer loyalty in omnichannel retailing using purchase behavior, socio-cultural factors, and learning techniques
Source: PLoS One. 2025 Aug 21;20(8):e0330338. doi: 10.1371/journal.pone.0330338 (PMC12370124; doi:10.1371/journal.pone.0330338)
Supplement: Attachment — Submitted filename: response PLOS ONE.docx [file pone.0330338.s001.docx]

The paper entitled „*Predicting Customer Loyalty in Omnichannel Retailing Using Purchase Behavior, Socio-Cultural Factors, and Learning Techniques*” is interesting and apply a few models based on modelling and prediction.

The topic is challenging in this era of relationship based on using AI and modelling, but in order to be publisehd the authors need to make some improvements:

-In the **Introduction**, the authors need to add text about the novelty of this paper, and the gap in the literature review and empirical comparing to their own study;

-To Literature review the authors need to add some updated sources for **Sentiment analysis**, for **BERT model**, **Evaluation Metrics** and also for **Hyperparameter Tuning,** there are no sources.

-The **Methodology** is well done and explained, but needs some improvements such as: for the **regression equation** determined by the authors must be added a clear explanation of the increasing/decreasing of the values for each independent variable and their influences on dependent variable how is explained (loyalty).

-Theoretical implication need to be more developed and the Practical ones, written in italic both, to be easy perceived by the readers, to be more explained for the categories implied: not only for retailers, but also for customers and society/police makers in the field.

Therefore, having these measures for improvements in view, the paper receives **major revision**.
