# Supplementary material for: Predicting customer loyalty in omnichannel retailing using purchase behavior, socio-cultural factors, and learning techniques
Source: PLoS One. 2025 Aug 21;20(8):e0330338. doi: 10.1371/journal.pone.0330338 (PMC12370124; doi:10.1371/journal.pone.0330338)
Supplement: Attachment — Submitted filename: Response to the Reviewers.docx [file pone.0330338.s004.docx]

**Title: Response to the Reviewers' Comments**

We would like to express our sincere gratitude to the esteemed reviewers for their valuable and constructive feedback. All comments have been thoroughly reviewed, and the necessary changes have been made to the article. Here, we present our responses to the reviewers' comments in detail. We hope that we have understood the valuable comments of the esteemed reviewers and have made the appropriate changes.

*********************************************************************************************

**Reviewer 1:**

We sincerely thank you for the valuable suggestions you provided, which have significantly contributed to improving our manuscript. We are truly grateful for the time and effort you dedicated to reviewing our work.

**1-In the Introduction, the authors need to add text about the novelty of this paper, and the gap in the literature review and empirical comparing to their own study**

Answer: Thank you for your valuable feedback. The requested item has been added to the introduction as follows:

Despite numerous studies on customer loyalty in multi-channel retail environments, significant gaps still exist in this area. Most previous research has focused on analyzing sales data and online customer behavior, addressing only limited factors such as purchase history and transaction data. However, a more accurate understanding of customer behavior requires analyzing multi-channel data and examining social, economic, and cultural variables that significantly influence purchasing decisions and customer loyalty. Additionally, many studies have been limited to transactional data and online customer behavior, with the impact of factors such as economic and cultural conditions on customer loyalty not being thoroughly investigated. Advanced machine learning models, such as Graph Convolutional Networks (GCNs) and Transformer models, can simulate complex and nonlinear relationships in data and provide more accurate predictions. However, the use of these models for predicting customer loyalty has not yet been extensively explored. Many existing studies on loyalty have focused primarily on loyalty drivers or predicting loyalty using sales data and online behavior analysis, often concentrating on one or two sales channels (such as physical stores or online platforms). In contrast, customer interactions with retailers occur across multiple channels, including websites, physical stores, and social media platforms. To address these research gaps, this study aims to develop and expand loyalty prediction models by considering not only transactional data but also social, cultural, economic, and emotional factors, RFM analysis, Customer Lifetime Value (CLV), churn rate, and customer behavior analysis—including shopping cart analysis, customer behavior dynamics, latent behavioral patterns, and sales data—to achieve more accurate predictions of loyalty.

While prior studies have explored customer loyalty using transactional or behavioral data, few have integrated socio-cultural, emotional, and macroeconomic variables into a unified predictive framework. Moreover, traditional models often neglect the dynamic nature of loyalty in omnichannel environments.From an empirical perspective, most previous research has relied on either survey-based data or limited-channel transactional records, often lacking large-scale, real-world data that includes cross-channel behaviors. In contrast, our study uses over 300,000 real omnichannel transactions from a multinational retailer, capturing both online and offline behavior across diverse geographic, cultural, and economic contexts.Furthermore, prior works rarely conduct loyalty prediction at both the individual and neighborhood levels simultaneously. Our dual-level modeling framework not only improves prediction accuracy but also enables practical CRM strategies tailored by customer segment and region, which is seldom addressed in existing empirical studies.This research addresses both theoretical and empirical gaps by combining sentiment analysis (via BERT), cultural dimensions (Hofstede), and economic indicators with advanced learning models such as GCN and Transformer. Unlike earlier studies that rely solely on RFM or CLV, our hybrid approach enhances prediction accuracy by modeling complex behaviors within a dynamic multichannel retail environment.

These variables can have a profound impact on customer purchase decisions, making it essential to simulate customer behavior more effectively by considering these factors. In addition to traditional models, this study employs advanced machine learning techniques, such as Graph Convolutional Networks (GCNs) and Transformer models, to analyze complex datasets and provide more precise loyalty predictions. These algorithms can simulate customer behavior with greater accuracy and offer optimized solutions for marketing strategies. To bridge the research gaps, an initial baseline model has been implemented to predict customer loyalty in a simplified manner. This baseline model excludes social, economic, and cultural variables, serving as a foundation for further model enhancements and refinements. This baseline model serves as a reference point for comparison with more advanced models that incorporate these additional factors. By combining these models, we can evaluate the impact of these factors on loyalty prediction accuracy and demonstrate how integrating them enhances prediction precision. In many cases, complex relationships exist within the stored organizational data, necessitating robust approaches to identify and analyze these intricate connections. While the use of machine learning techniques in predicting customer behavior and loyalty is increasing, this research gap presents new opportunities for applying advanced machine learning techniques such as Graph Convolutional Networks (GCNs), Transformer models. These techniques enable the analysis of complex datasets and more accurate customer loyalty predictions.

**********************************************************************************************************************2-To Literature review the authors need to add some updated sources for Sentiment analysis, for BERT model, Evaluation Metrics and also for Hyperparameter Tuning, there are no sources.**

Answer: We sincerely appreciate your highly valuable and pertinent feedback. Your requested suggestion has been graciously accepted and incorporated into the paper as follows:

In recent years, sentiment analysis has been enriched by deep learning techniques, particularly transformer-based models such as BERT (Bidirectional Encoder Representations from Transformers). BERT has demonstrated advanced performance in text classification tasks, including customer sentiment analysis in retail contexts
[134-136].

Evaluation metrics such as Accuracy, Precision, Recall, F1-Score, and Area Under the ROC Curve (ROC-AUC) are standard in classification problems and provide a comprehensive view of model performance
[137].

Hyperparameter tuning plays a critical role in optimizing model accuracy. Techniques such as Random Search and Particle Swarm Optimization (PSO) have been effectively applied to tune complex models in customer behavior studies [138-140].

[1]- Devlin, J., Chang, M. W., Lee, K., & Toutanova, K. (2019, June). Bert: Pre-training of deep bidirectional transformers for language understanding. In Proceedings of the 2019 conference of the North American chapter of the association for computational linguistics: human language technologies, volume 1 (long and short papers) (pp. 4171-4186).‏
[2]- Karabila, I., Darraz, N., EL-Ansari, A., Alami, N., & EL Mallahi, M. (2024). BERT-enhanced sentiment analysis for personalized e-commerce recommendations. Multimedia Tools and Applications, 83(19), 56463-56488
[3]- Geetha, M. P., & Renuka, D. K. (2021). Improving the performance of aspect based sentiment analysis using fine-tuned Bert Base Uncased model. International Journal of Intelligent Networks, 2, 64-69.‏
[4]- Sokolova, M., & Lapalme, G. (2009). A systematic analysis of performance measures for classification tasks. Information processing & management, 45(4), 427-437.‏
[5]- Lorenzo, P. R., Nalepa, J., Kawulok, M., Ramos, L. S., & Pastor, J. R. (2017, July). Particle swarm optimization for hyper-parameter selection in deep neural networks. In Proceedings of the genetic and evolutionary computation conference (pp. 481-488).‏
[6]- Soares, R. C., Silva, J. C., de Lucena Junior, J. A., Lima Filho, A. C., de Souza Ramos, J. G. G., & Brito, A. V. (2025). Integration of Bayesian optimization into hyperparameter tuning of the particle swarm optimization algorithm to enhance neural networks in bearing failure classification. Measurement, 242, 115829.‏
[7]- Agrawal, P. (2024, March). A survey on hyperparameter optimization of machine learning models. In 2024 2nd International Conference on Disruptive Technologies (ICDT) (pp. 11-15). IEEE.‏

***********************************************************************************************3-The Methodology is well done and explained, but needs some improvements such as: for the regression equation determined by the authors must be added a clear explanation of the increasing/decreasing of the values for each independent variable and their influences on dependent variable how is explained (loyalty)**

Answer: Thank you for your valuable feedback. The requested changes have been incorporated into the manuscript as follows:

The regression equation obtained via SEM indicates both the magnitude and direction of influence that each independent variable exerts on customer loyalty. The standardized path coefficients are interpreted as follows:

- **Customer Emotion (β = 0.26):** A one-unit increase in the emotional sentiment score—extracted via BERT from customer reviews—is associated with a 0.26 standard deviation increase in the loyalty score. This highlights emotional engagement as the strongest driver of loyalty in our model.
- **Customer Lifetime Value (β = 0.21):** Customers with higher CLV tend to be significantly more loyal. A one-unit increase in standardized CLV leads to a 0.21 increase in loyalty. This confirms the role of long-term customer profitability in shaping brand commitment.
- **RFM Score (β = 0.19):** This composite score reflects recency, frequency, and monetary value. Frequent and recent buyers contribute positively to loyalty. Each one-unit rise in RFM predicts a 0.19 unit rise in loyalty.
- **Customer Segment (β = 0.16):** Premium segments (as derived from purchasing patterns) show higher loyalty compared to new or occasional buyers.
- **Total Purchases (β = 0.12):** Although positively correlated, this variable has a relatively smaller effect. It may overlap with CLV and RFM, suggesting diminishing marginal returns from purchase count alone.
- **Customer Churn (β = -0.06):** This variable exhibits a negative relationship, indicating that an increase in churn probability decreases loyalty. Although the effect is modest, it is statistically significant and useful for early churn detection.

Together, these results offer a holistic view of loyalty as a function of **emotional, behavioral, and financial dimensions**. Notably, emotional factors (β = 0.26) have a stronger effect than pure purchase frequency (β = 0.12), highlighting the psychological aspect of loyalty in omnichannel contexts.

***********************************************************************************************4-Theoretical implication need to be more developed and the Practical ones, written in italic both, to be easy perceived by the readers, to be more explained for the categories implied: not only for retailers, but also for customers and society/police makers in the field.**

Answer: Thank you for your valuable feedback. The requested content has been added to the discussion and conclusion section as follows:

Practical Implementation for Retail Managers:

- Personalized Customer Interaction: Retailers can categorize customers based on loyalty scores and tailor their marketing messages for each group.
- Dynamic Pricing and Advertising: Real-time analysis of customer behavior enables retailers to adjust pricing and advertising strategies dynamically and purposefully.
- Improved Customer Experience: Analyzing customer behavior patterns and sentiments helps address their concerns and improve the shopping experience.
- Scenario-Based Decision-Making: By using reinforcement learning (Q-learning), businesses can simulate different economic and competitive scenarios and design effective strategies for retaining customers under various conditions.

Theoretically, this research contributes to the field by demonstrating the impact of combining socio-cultural, emotional, and economic indicators with traditional transactional features using an adaptive machine learning framework. It expands existing loyalty models by including neighborhood-level segmentation and dynamic sentiment analysis. From a practical perspective, the model offers key benefits for different stakeholders. Retailers, can optimize promotional strategies, inventory planning, and customer segmentation by using loyalty prediction scores. Customers,will experience improved personalization and satisfaction through adaptive recommendation systems informed by loyalty insights. Policy makers, can gain insights into socio-economic factors affecting consumer behavior, which can guide policy design in digital commerce and consumer protection. These stakeholder-focused benefits underline the model's relevance for both business application and policy development.

Therefore, integrating advanced machine learning techniques in predicting customer loyalty provides an innovative approach for multichannel retailers to strengthen customer loyalty and optimize performance. By combining social, economic, and behavioral data in predictive models, businesses can make data-driven decisions that enhance customer relationships, improve satisfaction, and ultimately increase profitability. Implementing these insights helps managers achieve sustainable growth in a competitive market and optimize their operational efficiency. Data analysis and the use of learning models always face challenges. In this study, due to the great diversity of data and the numerous features available, it is not unexpected that Emcom faced challenges such as adjusting and optimizing model parameters and preventing overfitting of the model. To deal with this problem, parameter tuning techniques such as PSO were used. This technique helped prevent overfitting and improved the performance of the model. Another challenge is the diversity and complexity of omnichannel data collected from different sales channels, such as physical and online stores. Therefore, great care must be taken in choosing a prediction model to increase the accuracy of the prediction. In this study, by using hybrid approaches, we have been able to achieve high accuracy in predicting loyalty. However, conducting this research comes with certain limitations. Some of these limitations include:

**********************************************************************************************************************************************************************************************

**Reviewer 2:**

**1-** **The paper mentions using a Kaggle dataset, but doesn't fully describe its limitations. A detailed description of the dataset's characteristics (sample size, geographic distribution, temporal coverage, etc.) and a discussion of potential biases is needed. The claim of "diverse demographics, geographic locations, product categories, and brands" needs substantiation with specific numbers and details. The fact that the data comes from a single, unnamed omnichannel retailer significantly limits the generalizability of the findings. The authors acknowledge this limitation, but a more thorough discussion of the potential biases introduced by this single-source data is needed.**

Answer: Thank you for your valuable feedback. The requested content has been added to the discussion and conclusion section as follows:

Although the statistics presented in Table 8 indicate an acceptable level of demographic and product diversity, several serious limitations must be considered:

**Single-source bias:** The data originates from a single multi-channel retailer. This brand (unnamed due to data restrictions) may have policies, discount strategies, user experience, and customer service practices that differ significantly from other retailers. As a result: Customer loyalty might stem not from general customer behavior but from brand-specific policies. The brand’s interaction style with customers (e.g., loyalty programs, exclusive discounts, employee behavior) could artificially inflate or deflate perceived loyalty. If the brand enjoys particular popularity or recognition in specific countries (e.g., India or the U.S.), the findings may not generalize to less well-known brands.

**Temporal bias:**The data only covers the past two years (2023–2024). These years were marked by specific economic and social events (such as global inflation, post-COVID recession, and rapid digital transformation). Therefore: Customer behavior may not represent long-term or stable trends. Rapid technological shifts (like the rise of AI in shopping) might have different effects in other timeframes.

**Brand-centric bias:** Despite the variety of brands in customer baskets, all interactions took place within the retailer’s platform. Therefore: Brands available only on this platform may have had a disproportionate impact on customer loyalty. Data related to specific brands outside this platform were not available.

**Lack of access to external behavioral variables:** Such as social media interactions, engagement with competitors, or exposure to external advertising. All interactions are system-internal and may not reflect the full picture of customer purchase behavior.

**Data imbalance:** Some sales channels, such as the mobile app, are underrepresented compared to the website and physical store, which could bias the model toward the dominant channel.

While the data offers a reasonable degree of diversity, the findings of this study should be generalized to other markets or industries with caution. Future research is recommended to incorporate data from multiple brands, industries, and geographic regions to improve the external validity of the model

*************************************************************************************************

**2-** **The description of data preprocessing is superficial. More detail is needed on how missing values were handled, how categorical variables were encoded, and the specific methods used for feature scaling and normalization. The creation of new variables like "Holiday Status" and the various impact scores requires a more rigorous explanation of the methodology and justification for the chosen methods. The lack of detail makes it difficult to assess the validity and reliability of these engineered features.**

Answer: Thank you for your valuable feedback. The requested changes have been incorporated into the manuscript as follows:

To manage the data, the following steps were performed:

1. Missing Data Handling: Median imputation was applied to numerical features (e.g., Age, Total Amount) due to its robustness against outliers. Categorical variables (e.g., Gender, Shipping Method) with missing values were assigned a placeholder category labeled "unknown" to avoid introducing bias. Outliers in numerical fields were detected and capped using the interquartile range (IQR) method.
2. Encoding & Transformation: Ordinal features such as "Income" and "Customer Segment" were numerically encoded while preserving their natural order (Low < Medium < High). Nominal features were one-hot encoded as appropriate. Customer feedback, originally in free-text format, was transformed into a 1–5 Likert scale based on sentiment analysis.
3. Feature Engineering: The Holiday Status variable was generated by extracting purchase dates and matching them with national, religious, and cultural holidays using Python’s holidays package (for the US, UK, and India), enabling detection of behavior changes on culturally significant days. Transaction timestamps were decomposed into day, month, weekday, and weekend/holiday flags for time-aware modeling. Address fields were parsed to extract urban density indicators (e.g., zip code clusters) for geographic behavioral analysis.
4. Impact Score Construction: Composite variables such as Social Impact Score and Cultural Impact Score were created using machine learning models (XGBoost, Random Forest) trained to predict transaction amounts or sentiment from demographic and contextual features. Feature importance weights were normalized and used to construct the scores, providing an interpretable data-driven measure of social and cultural influences.
5. Normalization:All numerical variables were standardized using StandardScaler (mean=0, std=1). Scaling was performed separately on training and test sets to prevent data leakage.
6. Justification & Limitations: Median imputation was chosen over mean imputation for its resilience to extreme values. Separate preprocessing for train and test sets ensured model validity. The use of exact transaction dates allowed accurate holiday detection. Although replacing missing categorical values with "unknown" may cause minor information loss, this was considered acceptable given the low missingness rate (<2%) and the model’s capacity to learn latent representations.

*************************************************************************************************

**3-**  **The authors acknowledge the limitations of generalizing findings to different markets or industries, but a more in-depth discussion of the specific factors that might limit generalizability is needed. The reliance on a single retailer's data significantly impacts the external validity of the study.**

Answer: Thank you for your valuable feedback. The requested changes have been incorporated into the manuscript as follows:

**Generalizability Limitations**

Although this study aimed to design a relatively comprehensive customer loyalty prediction model using data with demographic, geographic, and product diversity, full reliance on the data from a single multi-channel retailer poses a significant limitation in terms of generalizing the findings to other industries or markets. To clarify this issue, several specific characteristics of this retailer can be highlighted—factors that may limit the replicability of the findings in other contexts or industries:

**Discounting and Pricing Strategy**: The retailer under study may employ extensive discounts, strong loyalty programs, or region-based pricing strategies. In such cases, customer loyalty behavior is more likely driven by financial incentives rather than emotional or brand-driven loyalty. This pattern does not hold in non-price-sensitive industries (e.g., healthcare or insurance).

**Specific Product Mix**: The retailer offers a distinct product portfolio including electronics, clothing, food, books, and home decor. As a result, the observed loyalty patterns mostly reflect everyday or personal purchases—not long-lifecycle services such as banking, insurance, or education.

**Demographic Profile of the Retailer’s Customers**: Most customers fall within the 20–45 age range and belong to middle or upper-middle socioeconomic classes. Therefore, the recorded loyalty behaviors may reflect the habits of a younger, digitally oriented generation rather than traditional consumers or underserved populations.

**Digital Experience and Technical Infrastructure**: This retailer utilizes advanced recommender systems, a strong user interface design, and a robust mobile app. In industries that have not yet undergone digital transformation (e.g., local brick-and-mortar stores or rural markets), similar loyalty models may not function as effectively.

**Behavioral Model Trained on Intra-brand Stimuli**: All behavioral data (e.g., purchase history, browsing patterns, cart activity, and feedback) were generated within the ecosystem of the same brand. This means the model is sensitive only to interactions within that environment. Customer behavior in response to competitors or across multiple brands was not included.

Although the proposed model demonstrates high accuracy in analyzing customer loyalty for this specific retailer, its generalizability to other industries, B2B markets, service sectors, or small/non-digital businesses is limited due to its dependence on the retailer’s structure, product types, pricing policies, and customer demographics. Future studies should incorporate more diverse datasets across different industries (such as insurance, education, transportation, or financial services) and from multiple brands to enhance the external validity and applicability of the model.

*************************************************************************************************

**4-** **While the paper mentions using several machine learning models, the rationale behind choosing specific models (BERT, Reinforcement Learning, GCN, Transformer) and their combinations needs further justification. The authors should clearly articulate why these specific models are appropriate for this problem and how they address the limitations of previous research. A more detailed comparison of the strengths and weaknesses of each model in relation to the research question is necessary.**

Answer: Thank you for your valuable feedback. The requested changes have been incorporated into the manuscript as follows:

The selection of BERT, GCN, Transformer, and Reinforcement Learning (Q-Learning) models in this study was driven by the need to address the multidimensional and complex nature of customer loyalty within a multi-channel retail environment. Unlike many purely behavioral phenomena, loyalty is a combination of emotional, social, economic, and behavioral factors that manifest across structured, unstructured, and network data. Traditional models such as Logistic Regression, RFM, or even XGBoost lack the capacity to effectively process this diversity.

BERT was chosen for its ability to analyze unstructured customer feedback text, extracting sentiments and understanding contextual and implicit language nuances with high accuracy. This capability surpasses older methods like TF-IDF or LSTM, which tend to be more superficial and lack deep contextual comprehension.

GCN (Graph Convolutional Network) was employed to model the network structure of customer interactions, such as participation in campaigns and shared reviews. GCN excels at identifying hidden network patterns and peer influences, which classical models usually ignore by treating customers as independent entities.

Transformer models were selected to handle sequential data, such as purchase histories and session logs, due to their superior performance in capturing long-term temporal and textual dependencies through self-attention mechanisms. Unlike LSTM models, which suffer from the vanishing gradient problem and memory loss over long sequences, Transformers maintain better contextual awareness.

Reinforcement Learning, specifically Q-Learning, was utilized to simulate marketing decision scenarios and customer purchase behaviors, enabling the modeling of long-term reward-based decision making. This approach overcomes the static nature of traditional predictive models by incorporating the dynamic interaction between decisions and loyalty outcomes.

By combining these models, the analysis extends beyond merely relying on past customer behaviors. It incorporates psychological dimensions via sentiment analysis with BERT, social relationships and network effects through GCN, and dynamic customer decision-making in response to campaigns through Q-Learning. This comprehensive approach addresses the key limitations of earlier studies that focused narrowly on RFM, CLV, or simple LSTM models.

Additionally, model design carefully considered the alignment between data type and model architecture. For instance, BERT was favored over LSTM/GRU for textual feedback due to its superior understanding of complex sentences, while Transformer was chosen for sequence modeling because of its more accurate self-attention mechanism compared to traditional RNNs.

From an implementation perspective, all models were trained on a combined loyalty index derived from SEM, and their outputs were integrated using a weighted ensemble approach that assigns weights based on each model’s accuracy. This ensemble technique enhanced overall prediction accuracy and reduced forecast variability.

In summary, the choice of these architectures was guided not only by technical compatibility with data types but also by the specific needs of the problem and the aim to fill methodological gaps in the existing customer loyalty literature.

*************************************************************************************************

**5-** **The paper lacks detail on hyperparameter tuning for the various models. How were the hyperparameters selected and optimized? Were techniques like cross-validation used? This information is crucial for assessing the reproducibility and reliability of the results.**

Answer: Thank you for your valuable feedback. The requested changes have been incorporated into the manuscript as follows:

To ensure a scientifically rigorous and robust hyperparameter tuning process, a systematic approach was adopted. For each hyperparameter configuration sampled via Random Search or optimized through Particle Swarm Optimization (PSO), **5-fold cross-validation** was employed on the training data to reliably evaluate model performance and mitigate the risk of overfitting. The evaluation metric used for selection was the **mean F1-score** across all validation folds, providing a balanced measure of precision and recall, which is particularly suitable for customer behavior classification tasks with potentially imbalanced classes.

The hyperparameter search spaces were carefully defined based on domain knowledge and relevant literature to cover meaningful value ranges while avoiding excessively large or irrelevant domains. PSO parameters such as population size, inertia weight, and cognitive and social coefficients were set following standard guidelines to balance exploration and exploitation in the search process. This combination of advanced optimization techniques with robust cross-validation provides a scientifically sound and reliable framework for hyperparameter tuning, ensuring that the final models are both well-optimized and generalizable.

*************************************************************************************************

**6-** **The paper uses several evaluation metrics (Accuracy, ROC-AUC, Precision, Recall, F1-score), but a more thorough discussion of the choice of these metrics and their interpretation in the context of the problem is needed. Why were these specific metrics chosen, and what are their limitations? The authors should also discuss the potential for class imbalance in the dataset and how this was addressed.**

Answer: Thank you for your valuable feedback. The requested changes have been incorporated into the manuscript as follows:

To assess classification performance, we used Accuracy, Precision, Recall, F1-Score, and ROC-AUC. These were selected to provide a comprehensive performance view. **Accuracy** provides an overall measure of correct predictions but can be misleading when classes are imbalanced.**Precision** measures the proportion of correctly identified positive cases among all predicted positives, which is critical to minimize false positives. **Recall** (Sensitivity) assesses the ability to detect actual positive cases, important to avoid missing loyal customers. **F1-Score** offers a harmonic mean of Precision and Recall, balancing the trade-off between false positives and false negatives. **ROC-AUC** reflects the model’s capability to discriminate between classes across all classification thresholds. Given the potential class imbalance in the dataset (i.e., the proportion of loyal customers is smaller), we applied the Synthetic Minority Oversampling Technique (SMOTE) to the training data to balance class distribution and improve the reliability of the evaluation metrics.

*************************************************************************************************

**7-**  **The comparison with baseline models (RFM and CLV) is insufficient. The paper needs to provide a more detailed description of the baseline models used and how they were implemented. A more rigorous comparison of the proposed model's performance against these baseline models is necessary to demonstrate the added value of the proposed approach.**

Answer: Thank you for your valuable feedback. The requested changes have been incorporated into the manuscript as follows:

Based on the literature review and as far as we know, research has thus far focused on examining the factors affecting loyalty either through classic and traditional loyalty models such as RFM and CLV, or traditional machine learning models like XGBoost and LSTM to predict loyalty. Therefore, in this section, the performance of the proposed method was compared with traditional models. The limitations of traditional and classic models can be summarized as follows:

- Traditional customer loyalty prediction methods, such as RFM (Recency, Frequency, Monetary) models and CLV (Customer Lifetime Value), mainly rely on transactional data and overlook economic, social, and cultural factors. These methods have low accuracy in dynamic market conditions and cannot predict sudden changes in customer behavior.
- Traditional machine learning models like XGBoost and LSTM, while offering higher accuracy than RFM, are still limited to historical data and do not take social interactions and environmental changes into account.

In order to validate the methodological contribution of this research and assess the impact of economic, social, and cultural variables on customer loyalty prediction, a comprehensive comparative analysis was conducted. In this analysis, the proposed Adaptive GCN-Transformer hybrid model was compared with baseline models (which did not incorporate economic, social, cultural variables, customer sentiments, and churn rates) as well as traditional customer loyalty prediction methods. The baseline model was developed by combining two traditional loyalty estimation approaches—RFM (Recency, Frequency, Monetary) analysis and CLV (Customer Lifetime Value)—into a single composite scoring system. This combined model relied solely on behavioral and transactional data, without incorporating any socio-economic, emotional, or contextual features. The combination of CLV and RFM into a unified baseline model is not considered problematic, as both are based on transactional data yet reflect different but complementary dimensions of customer behavior—CLV focusing on long-term financial value, and RFM capturing short-term behavioral engagement. In the first step, a baseline model was developed that relied only on customers' behavioral and transactional data, without utilizing economic, social, cultural, or emotional variables. To establish a reliable benchmark and compare the performance of the proposed model, we implemented two well-established customer loyalty models: Recency-Frequency-Monetary (RFM) analysis and Customer Lifetime Value (CLV).

**RFM Model Implementation:** RFM analysis quantifies customer engagement based on three key behavioral indicators:

- **Recency (R):** The number of days since the customer’s last purchase.
- **Frequency (F):** The total number of purchases made during the observation period.
- **Monetary (M):** The total monetary value of the customer’s purchases in the same period.

Each dimension was computed for all customers using a 12-month observation window. The customers were then assigned a score from 1 to 5 for each dimension using **quintile-based binning**. The final RFM score was calculated by applying weights to each component to reflect their relative importance in loyalty prediction, as follows:

$$RFM Score=M\times0.3+F\times0.3+R\times0.4$$

Customers with higher overall RFM scores were considered more likely to be loyal.

**CLV Model Implementation:** Customer Lifetime Value (CLV) was estimated using the formula:

$$CLV=Expected Customer Lifespan\times Purchase Frequency\times Average Order Value$$

- **Average Order Value (AOV)** was calculated by dividing total revenue by total number of purchases.
- **Purchase Frequency** was computed as the number of purchases divided by the customer’s tenure.
- **Expected Customer Lifespan** was estimated using churn probabilities predicted via logistic regression.

The CLV values were then normalized and used as a proxy for the long-term economic value of each customer.

These models served as baseline methods against which the performance of the proposed hybrid approach was evaluated. The proposed model, which was designed by combining multichannel transactional data with economic, social, cultural variables, customer sentiments, and churn rates, was evaluated. The purpose of comparing these models was to assess the extent to which these additional features could improve prediction accuracy and overall model performance. The results of this comparison highlighted the importance of these variables in enhancing the accuracy of customer loyalty predictions and demonstrated that incorporating them could lead to a deeper analysis of customer loyalty. The performance of this baseline model was evaluated using standard metrics such as Accuracy, ROC-AUC (Area Under the Receiver Operating Characteristic Curve), Precision, Recall, and F1-Score, with the results presented in Table 16. The evaluation results showed that the proposed model achieved significant improvements across all performance metrics compared to the baseline model, confirming the critical role of these additional features in improving customer loyalty prediction accuracy. The comparative analysis is presented in Figures 9 and 10 and Table 18.

| **Table 18- Evaluation Criteria of the Baseline Model** | | |
| --- | --- | --- |
| **Evaluation Criteria** | **Baseline Model** | |
|  | **Predicting Customer** | **Neighborhood** |
| Accuracy | 0.68 | 0.72 |
| ROC-AUC | 0.7 | 0.82 |
| Precision | 0.8 | 0.61 |
| Recall | 0.79 | 0.58 |
| F1-Score | 0.72 | 0.6 |

The comparison of the baseline model's performance with other advanced models for predicting loyalty for both customers and neighborhoods is clearly illustrated in Figure 9. The results presented in Table 16 and Figures 9,10 indicate that incorporating economic, social, cultural factors, customer sentiments, and churn rates significantly contributes to improving loyalty prediction.

|  | Accuracy | ROC-AUC | Precision | Recall | F1-Score |
| --- | --- | --- | --- | --- | --- |
| Mode1 | 0.68 | 0.7 | 0.8 | 0.79 | 0.72 |
| Model 3 | 0.97 | 1 | 0.96 | 0.97 | 0.97 |
| Model 2 | 0.94 | 0.99 | 0.95 | 0.93 | 0.94 |
| Model 5 | 0.98 | 0.98 | 0.97 | 1 | 0.98 |
| Model 6 | 0.9 | 0.9 | 0.9 | 0.9 | 0.99 |
| Model 8 | 0.96 | 0.96 | 0.96 | 0.96 | 0.96 |

**Figure 9 - Comparison of the Baseline Model and Advanced Models in Predicting Customer Loyalty**

|  | Accuracy | ROC-AUC | Precision | Recall | F1-Score |
| --- | --- | --- | --- | --- | --- |
| Mode1 | 0.72 | 0.82 | 0.61 | 0.58 | 0.6 |
| Model 3 | 0 | 0 | 0 | 0.5 | 0.74 |
| Model 7 | 0.64 | 0.88 | 0.71 | 0.58 | 0.64 |
| Model 4 | 0.86 | 0.94 | 0.84 | 0.94 | 0.86 |
| Model 5 | 0.84 | 0.86 | 0.73 | 0.58 | 0.64 |
| Model 6 | 0.89 | 0.99 | 0.89 | 1 | 0.89 |

**Figure 10 - Comparison of the Baseline Model and Advanced Models in Predicting Neighborhood Loyalty**

Model 1: Baseline Model

Model 2: Classification Model and Autoencoder

Model 3: CNN

Model 4: Autoencoder

Model 5: PCA & Random Forest

Model 6: XGBoost & LSTM

Model 7: PSO and LSTM

Model 8: Adaptive GCN-Transformer Hybrid

The proposed hybrid model outperformed the baseline models by 19% in F1-score and 23% in ROC-AUC, indicating the added value of incorporating socio-cultural and emotional data. Some of the advantages that make the proposed method superior to traditional and classical models include the following:

- Comparing the performance charts, the superiority of the proposed method in terms of loyalty prediction accuracy is clearly visible. The proposed model has an 8% higher accuracy and a 15% improvement in F1-Score compared to the baseline method, indicating a reduction in prediction error and an improvement in performance.
- The use of Graph Convolutional Networks (GCN) for analyzing customer relationships and discovering hidden patterns among them.
- Modeling customer behavioral changes with Transformer, which allows for learning purchase time patterns.
- Combining transactional data with economic, social, cultural variables, and customer behaviors like sentiments, etc., which aids in more accurate loyalty prediction.
- • Utilizing reinforcement learning (Q-Learning) to simulate different scenarios and assess the impact of environmental factors on customer loyalty.

Given the acceptable results of the proposed model and its advantages, it can be stated that this model helps retail companies optimize their marketing and customer management strategies through the following:

- Early identification of customer loyalty decline and offering targeted discounts before they churn.
- Analyzing the economic impacts (such as inflation and unemployment) on customer buying behavior and adjusting pricing strategies accordingly.
- Optimizing product recommendation systems based on changes in customer behavior.

Therefore, it can be said that the proposed Adaptive GCN-Transformer Hybrid model, by combining deep learning, graph analysis, and advanced language models, has achieved better performance compared to traditional methods. This approach has significantly improved customer loyalty prediction and serves as a powerful tool for marketing managers and multichannel businesses.

*************************************************************************************************

**8-** **The use of SEM to determine weights for combining different indicators into a loyalty score requires more explanation. The authors should provide more detail on the SEM model specification, the model fit indices, and the justification for the chosen weighting scheme. The interpretation of the SEM results needs to be more thorough and nuanced.**

Answer: Thank you for your valuable feedback. The requested changes have been incorporated into the manuscript as follows:

**Results Analysis for SEM in Combination with Loyalty-Influencing Variables**

In this study, Structural Equation Modeling (SEM) was used to analyze data and model complex relationships between various variables. As observed in the literature review section, this method is particularly effective in analyzing complex and multidimensional relationships between variables in behavioral and customer studies. SEM is capable of simultaneously modeling direct and indirect relationships between variables, making it suitable for analyzing customer behavior and predicting customer loyalty. This method is especially useful when complex relationships and high correlations between variables need to be analyzed.

In this study, SEM was used to model and analyze relationships among various variables, including customer loyalty, customer emotions, purchase timing, purchase trends, purchase amount, and RFM. This method is also beneficial when dealing with high correlations between variables, as it allows for simultaneous modeling of complex relationships. First, the correlation matrix between observable indicators (Table 12) was examined. The results showed that some of the indicators had significant correlations with each other. However, to accurately manage potential multicollinearity and construct stable variables, the SEM method was utilized. In the modeling process, observable indicators (including Customer Segment, Customer Lifetime Value, Customer Emotion, Customer Churn, Total Purchases, and RFM Indicators) were modeled as latent variables. The combination of these indicators was done in a data-driven manner based on the factor loadings extracted from the measurement model. Therefore, the weighting of the indicators was based on precise statistical analyses, minimizing the potential for subjective errors in variable combination.

The correlation chart shown in Table 12 illustrates the strong relationships and high correlations between variables such as RFM, customer emotions, customer lifetime value, and total purchases. Specifically, it is observed that RFM and customer emotions are highly correlated with each other, which can directly impact customer loyalty prediction. While these variables are significantly correlated, the use of SEM effectively managed these correlations. By modeling both direct and indirect relationships, SEM not only examined the impacts of high correlations but also simultaneously and integrally modeled these complex relationships. This feature of SEM enabled accurate and effective analysis of relationships among the variables.

| **Table 12-Correlation Matrix of Customer Features** | | | | | | |
| --- | --- | --- | --- | --- | --- | --- |
|  | **Customer Segment** | **Customer Lifetime Value** | **Customer Emotion** | **Customer Churn** | **Total Purchases** | **RFM** |
| **Customer Segment** | 1.00 | 0.82 | 0.76 | 0.79 | 0.81 | 0.85 |
| **Customer Lifetime Value** | 0.82 | 1.00 | 0.78 | 0.74 | 0.80 | 0.88 |
| **Customer Emotion** | 0.76 | 0.78 | 1.00 | 0.77 | 0.75 | 0.79 |
| **Customer Churn** | 0.79 | 0.74 | 0.77 | 1.00 | 0.78 | 0.80 |
| **Total Purchases** | 0.81 | 0.80 | 0.75 | 0.78 | 1.00 | 0.82 |
| **RFM** | 0.85 | 0.88 | 0.79 | 0.80 | 0.82 | 1.00 |

One of the main challenges in this study was the high correlations between various variables. Specifically, RFM and customer emotions had high correlations with other variables, which could have complex and interactive effects on customer loyalty prediction. SEM addressed this issue by using advanced path analysis and factor analysis techniques. In SEM, factor modeling was used to analyze the relationships between observed and latent variables, and weights and factor loadings were calculated. Additionally, the structural model of SEM examined the relationships between latent variables. SEM is particularly a powerful tool for analyzing data with high correlations and complexity.

According to article [129], to calculate the weights in the customer loyalty model, a measurement model is first created. In this model, customer loyalty is defined as a latent variable that is measured by a set of observed indicators. These indicators include variables such as Customer Emotion, Customer Lifetime Value (CLV), Total Purchases, RFM, and Churn Probability, all of which are directly related to customer loyalty.

Next, using structural equation modeling (SEM) software such as AMOS, SmartPLS, or Mplus, the designed model is executed. These software tools calculate the Factor Loading for each indicator. These factor loadings represent the impact of each indicator on the latent variable of customer loyalty. These values range from 0 to 1 or can occasionally be negative. These Factor Loading are presented in Table 13

In the third step, to normalize these factor loadings and calculate the final weights, the factor loadings are first summed (using absolute values for negative values if necessary). Then, each factor loading is divided by the total sum of the factor loadings to determine the weight of each indicator. These calculated weights are presented in Table 13.

| **Table 13 - Weights of Composite Variables for Loyalty** | | |
| --- | --- | --- |
| Weight | Factor Loading | Variable |
| 0.26 | 0.8 | Customer Emotion |
| 0.16 | 0.5 | Customer Segment |
| 0.06 | -0.2 | Customer Churn |
| 0.19 | 0.6 | RFM |
| 0.21 | 0.65 | Customer Lifetime Value |
| 0.12 | 0.4 | Total Purchases |

The sum of the weights should be exactly 1 or very close to 1. Finally, using these weights, a Loyalty Score is calculated, which is a composite score based on the different indicators and their corresponding weights. The final formula for calculating loyalty will be as follows:

$$Loyalty=0.26\times Customer Emotion+0.16\times Customer Segment+0.06\times Customer Churn+0.19\times RFM+0.21\times Customer Lifetime Value+0.12\times Total Purchases$$

The regression equation obtained via SEM indicates both the magnitude and direction of influence that each independent variable exerts on customer loyalty. The standardized path coefficients are interpreted as follows:

- **Customer Emotion (β = 0.26):** A one-unit increase in the emotional sentiment score—extracted via BERT from customer reviews—is associated with a 0.26 standard deviation increase in the loyalty score. This highlights emotional engagement as the strongest driver of loyalty in our model.
- **Customer Lifetime Value (β = 0.21):** Customers with higher CLV tend to be significantly more loyal. A one-unit increase in standardized CLV leads to a 0.21 increase in loyalty. This confirms the role of long-term customer profitability in shaping brand commitment.
- **RFM Score (β = 0.19):** This composite score reflects recency, frequency, and monetary value. Frequent and recent buyers contribute positively to loyalty. Each one-unit rise in RFM predicts a 0.19 unit rise in loyalty.
- **Customer Segment (β = 0.16):** Premium segments (as derived from purchasing patterns) show higher loyalty compared to new or occasional buyers.
- **Total Purchases (β = 0.12):** Although positively correlated, this variable has a relatively smaller effect. It may overlap with CLV and RFM, suggesting diminishing marginal returns from purchase count alone.
- **Customer Churn (β = -0.06):** This variable exhibits a negative relationship, indicating that an increase in churn probability decreases loyalty. Although the effect is modest, it is statistically significant and useful for early churn detection.

Together, these results offer a holistic view of loyalty as a function of emotional, behavioral, and financial dimensions. Notably, emotional factors (β = 0.26) have a stronger effect than pure purchase frequency (β = 0.12), highlighting the psychological aspect of loyalty in omnichannel contexts.

This method for calculating the weights and combining them to calculate the Loyalty Score provides a well-documented and precise approach for assessing customer loyalty based on various criteria. The weights obtained from SEM indicate the significant and meaningful impacts of variables such as customer emotions, customer lifetime value, and RFM on customer loyalty. The weights derived from the SEM model for each variable represent its relative importance in predicting customer loyalty. These weights were obtained through path analysis and factor modeling in SEM, reflecting the direct and indirect effects of the variables on each other. Specifically, the variables of customer emotions and RFM showed a greater impact on customer loyalty, which are directly related to customers' buying behavior and preferences.

After performing the SEM analysis, the test results indicate that the SEM model has effectively modeled the complex relationships between variables and managed the high correlations among them. In this study, the model fit test results and fit indices such as Chi-Square, RMSEA, CFI, and TLI show a good fit of the model and its high capacity to analyze complex relationships. Chi-Square = 1.87, RMSEA = 0.05, CFI = 0.98, and TLI = 0.97, all indicating an excellent fit of the SEM model and its high ability to analyze complex relationships. These results clearly show that the SEM model has correctly analyzed the relationships between various variables and has been able to manage the high correlations among them. In other words, the model fit results (presented in the results section) show that the model has a good fit (with suitable values for CFI, TLI, RMSEA, and SRMR), and there is no indication of severe multicollinearity among the observed variables. This ensures the validity and reliability of the indicator combination for measuring latent variables. These weights indicate that Customer Emotion and RFM have a greater influence on customer loyalty, which aligns with the assumptions of the structural equation modeling (SEM) and previous analyses. Therefore, by using SEM and conducting a thorough analysis of the relationships between variables, this study has effectively managed the high correlations between variables and modeled the complex relationships. The results of the tests and model fit indices demonstrate the high accuracy of the SEM model in predicting customer loyalty and analyzing the complex variables related to it. Additionally, the weights obtained from the model show significant and proportional effects of various variables in predicting customer loyalty.

The factor loadings and corresponding weights extracted through SEM provide interpretable insights into the influence of each observed variable on customer loyalty. Specifically, Customer Emotion received the highest positive weight (0.26), indicating that emotional engagement has the strongest association with loyalty. Similarly, RFM (0.19) and Customer Lifetime Value (0.21) also demonstrate strong positive contributions, reflecting the importance of purchasing behavior and long-term value in loyalty formation. On the other hand, Customer Churn received a small negative weight (–0.06), consistent with its inverse relationship to loyalty. These weightings, derived from validated SEM path models, confirm the theoretical assumption that loyalty is a multidimensional construct shaped by both behavioral and attitudinal factors. They also provide empirical justification for the structure of the Loyalty Score used as the target variable in the predictive modeling phase.

*************************************************************************************************

**9-** **The paper needs to provide more detail on the experimental setup, including the software and hardware used, to ensure the reproducibility of the results. The code or a detailed description of the implementation should be made available.**

Answer: Thank you for your valuable feedback.

The implementation was carried out using Python 3.10, and the experiments were conducted primarily in the Google Colaboratory Pro environment equipped with an NVIDIA Tesla T4 GPU (16GB VRAM). Additional local preprocessing and testing were performed on a Windows 11 machine with an Intel Core i7 processor, 16GB RAM, and an NVIDIA 2GB GPU.
A variety of libraries were employed including TensorFlow 2.12, Keras, PyTorch, Scikit-learn 1.3, and XGBoost. The modeling pipeline comprised the following steps:

1. **Data preprocessing**: Missing value imputation, outlier detection using the IQR method, z-score normalization, and one-hot encoding of categorical variables.
2. **Feature engineering**: Generation of new features such as customer segmentation, holiday labels, macroeconomic indicators (e.g., inflation, unemployment), and sentiment scores extracted using BERT-based language models.
3. **Loyalty scoring**: Latent loyalty scores were computed using structural equation modeling (SEM) and factor loadings, as described in Section X.
4. **Model training**: Multiple models were trained and optimized including the proposed Adaptive GCN-Transformer hybrid model, LSTM, XGBoost, Random Forest, and CNN. Hyperparameter tuning was performed using grid search where applicable.
5. **Evaluation**: Models were evaluated using five standard metrics—Accuracy, Precision, Recall, F1-score, and ROC-AUC.
   The entire modeling process is illustrated in Figure 1 and further detailed in Algorithm 1. Parameter configurations and training settings are summarized in Tables 6, 10, and 13.
   Although the source code is not publicly shared, the methods, data handling procedures, and parameter values have been described in sufficient detail to support reproducibility by independent researchers.

*************************************************************************************************

**10-** **The interpretation of the results needs to be more critical and nuanced. The authors should discuss the limitations of the findings and potential sources of error. The discussion should also address the implications of the findings for both researchers and practitioners.**

Answer: Thank you for your valuable feedback. The requested changes have been incorporated into the manuscript as follows:

**Critical Interpretation and Limitations**

Data analysis and the use of learning models always face challenges. In this study, due to the great diversity of data and the numerous features available, it is not unexpected that Emcom faced challenges such as adjusting and optimizing model parameters and preventing overfitting of the model. To deal with this problem, parameter tuning techniques such as PSO were used. This technique helped prevent overfitting and improved the performance of the model. Another challenge is the diversity and complexity of omnichannel data collected from different sales channels, such as physical and online stores. Therefore, great care must be taken in choosing a prediction model to increase the accuracy of the prediction. In this study, by using hybrid approaches, we have been able to achieve high accuracy in predicting loyalty.

While the proposed hybrid model demonstrated high predictive performance across multiple metrics, a nuanced interpretation reveals several insights and limitations. For instance, although the GCN-Transformer architecture captured relational and temporal patterns effectively, its performance showed slight variability across customer segments—particularly in distinguishing between regular and premium customers with similar purchase histories but different emotional scores. Furthermore, certain variables such as cultural impact and economic factors (e.g., inflation) may have delayed or indirect effects not fully captured in the model’s timeframe. Additionally, while sentiment scores derived from customer reviews contributed significantly to accuracy, these scores can be influenced by linguistic bias or platform-specific noise. The reliance on data from a single omnichannel retailer limits the generalizability of the findings. Despite the dataset’s geographic and demographic diversity, brand-specific promotions, loyalty programs, and customer experience policies may not reflect broader retail patterns. Future studies should replicate this framework using multi-retailer or cross-industry datasets.However, conducting this research comes with certain limitations. Some of these limitations include:

1. A more in-depth examination of the impact of cultural and social variables could be conducted.
2. Deep learning models require more data and longer processing times, which can be challenging in some cases.
3. Using alternative methods such as reinforcement learning requires up-to-date data, and in some cases, it may lead to inaccurate estimates.
4. There is a need to test the model with real business data instead of using public datasets like those on Kaggle.
5. Despite the high accuracy in predicting customer loyalty using the Q-Learning model, it is important to note that this model may encounter errors in more complex conditions or with low-quality data. For example, in scenarios where social or economic information is incomplete, the results may not reflect accurate changes. Additionally, using reinforcement learning methods like Q-Learning requires time-sensitive and up-to-date data, which may not always be available. While Q-Learning is a powerful method for predicting customer loyalty, it should be emphasized that this approach depends on reliable and comprehensive data. If environmental information is not properly simulated or if incomplete data is used, predictions may have lower accuracy.

In this study, the dataset used is extracted from an omnichannel retailer operating across various countries and cities, containing a wide range of customer profiles in terms of demographic and geographic characteristics. The geographic coverage of the sample is diverse, including customers from different cities and countries, which not only enriches the dataset but also enhances the generalizability of the findings to different contexts. Additionally, the sample includes key demographic variables such as age, income, gender, and customer segments, which help provide a more comprehensive representation of customer behavior in an omnichannel retail environment. However, while the geographic diversity of this data strengthens the external validity of the findings, it is important to acknowledge that the data comes from a single retailer. This may introduce brand-specific biases that may not be fully generalizable to other retail contexts. Moreover, although factors such as inflation and unemployment rates have been incorporated to consider broader economic and cultural conditions, the absence of the target variable (customer loyalty) means that the findings are based on an indirect index. Given the complexity of customer loyalty, further research across multiple retailers, industries, and countries is necessary to validate the proposed models and enhance their generalizability and external validity.

Implications for Research and Practice

- For researchers, this study suggests a path forward in developing integrated loyalty models that blend behavioral, emotional, and contextual variables. The effectiveness of combining graph-based learning with temporal attention mechanisms opens new directions for modeling consumer behavior more holistically.
- For practitioners, particularly marketing analysts and retail strategists, the model provides actionable insights. By identifying loyalty drivers with higher precision, businesses can personalize offerings, predict churn more effectively, and allocate resources toward high-value customer segments.
- For policy makers, the inclusion of macroeconomic and socio-cultural factors highlights the broader context in which loyalty emerges. Understanding these patterns can inform digital commerce policy, consumer rights strategies, and regional market development initiative
