# Supplementary material for: Predicting customer loyalty in omnichannel retailing using purchase behavior, socio-cultural factors, and learning techniques
Source: PLoS One. 2025 Aug 21;20(8):e0330338. doi: 10.1371/journal.pone.0330338 (PMC12370124; doi:10.1371/journal.pone.0330338)
Supplement: Attachment — Submitted filename: response PLOSONE 24.07.2025.docx [file pone.0330338.s003.docx]

The paper entitled „*Predicting Customer Loyalty in Omnichannel Retailing Using Purchase Behavior, Socio-Cultural Factors, and Learning Techniques*” is interesting and apply a few models based on modelling and prediction.

To be publisehd the authors have made all the proposed improvements:

-In the **Introduction**, the authors added the text about the novelty of this paper, and the gap in the literature review and empirical comparing to their own study;

-To Literature review was imporoved by adding updated sources for **Sentiment analysis**, for **BERT model**, **Evaluation Metrics** and also for **Hyperparameter Tuning;**

-In the **Methodology**, for the **regression equation** were added clear explanatiosn of the increasing/decreasing of the values for each independent variable and their influences on dependent variable (loyalty).

-Theoretical implications and the Practical ones, were developed and better explained for the categories implied: not only for retailers, but also for customers and society/policy makers in the field.

Therefore, having these measures for improvements in view, and the improvements made by the authors, the paper receives **acceptance of the paper to be published**.
